# T62. COMPARISON OF NEUROCOGNITIVE FUNCTIONS IN FIRST-EPISODE SCHIZOPHRENIA PATIENTS, NON-PSYCHOTIC SIBLINGS, AND INDIVIDUALS AT CLINICAL HIGH-RISK FOR PSYCHOSIS

**DOI:** 10.1093/schbul/sby016.338

**Published:** 2018-04-01

**Authors:** On Ki Chu, Wing Chung Chang, Hoi Ching Lee, Suet In Chan, Sanyin Chiu, Lai Ming Hui, Kit Wa Chan, Ho Ming Lee, Yi Nam Suen, Eric Chen

**Affiliations:** The University of Hong Kong

## Abstract

**Background:**

Neurocognitive impairment is a core feature of schizophrenia, and has been observed among healthy non-psychotic siblings of schizophrenia patients as well as individuals at clinical high-risk (CHR) for psychosis. Thus far, few studies have directly contrasted neurocognitive performance between non-psychotic siblings and CHR samples. Potential differential patterns of neurocognitive deficits among schizophrenia patients, familial high-risk and CHR samples remain to be clarified. This study aimed to compare neurocognitive functions among first-episode schizophrenia (FES) patients, their non-psychotic siblings, CHR individuals, and healthy controls.

**Methods:**

FES patients (n=69, mean age=25.3) and CHR individuals (n=97, mean age=21.1) without family history of psychosis were recruited from a territory-wide specialized early intervention service for psychosis in Hong Kong. A group of non-psychotic siblings of FES patients (n=50, mean age=25.4) and healthy controls (HC) (n=68, mean age=24.5) were also recruited. A standardized battery of neurocognitive tests encompassing working memory, processing speed, executive function, visual memory, verbal learning, and sustained attention was administered. Group differences were examined using analysis of covariance (ACOVA) with Bonferroni correction applied for statistical significance (P<0.008), controlling for age and years of education.

**Results:**

Compared with HC, FES patients exhibited significantly poorer performance across all neurocognitive domains (Hedges g ranged: 0.48–1.73), while CHR individuals demonstrated significantly worse neurocognitive functioning in all domains (Hedges g ranged: 0.53–1.15) but sustained attention. Non-psychotic performed significantly worse than HC in executive function (Hedges g=0.63, p<0.001), visual memory (Hedges g=0.57, p=0.002), verbal learning (Hedges g=0.52, p=0.001), and working memory (Hedges g=0.37, p=0.003). Among four groups, FES patients displayed the most severe neurocognitive impairment. The pattern of neurocognitive dysfunction was similar between CHR and non-psychotic sibling groups, except for processing speed, of which CHR individuals demonstrated greater degree of impairment than siblings in digit symbol coding test (p<0.001).

**Discussion:**

Our results indicate a gradient of neurocognitive impairment across FES, CHR and non-psychotic sibling samples, reflecting differential degrees of psychosis liability. Processing speed, as measured by digit symbol coding test, demonstrated the highest discriminant utility in discriminating CHR from familial high-risk individuals. Our findings thus confirm the critical role of neurocognitive dysfunction as a reliable risk indicator and an endophenotype for schizophrenia and related psychoses.

